# Supplementary material for: PER2 Circadian Oscillation Sensitizes Esophageal Cancer Cells to Chemotherapy
Source: Biology (Basel). 2021 Mar 26;10(4):266. doi: 10.3390/biology10040266 (PMC8065910; doi:10.3390/biology10040266)
Supplement: Supplementary file 1 [file biology-10-00266-s001.pdf]

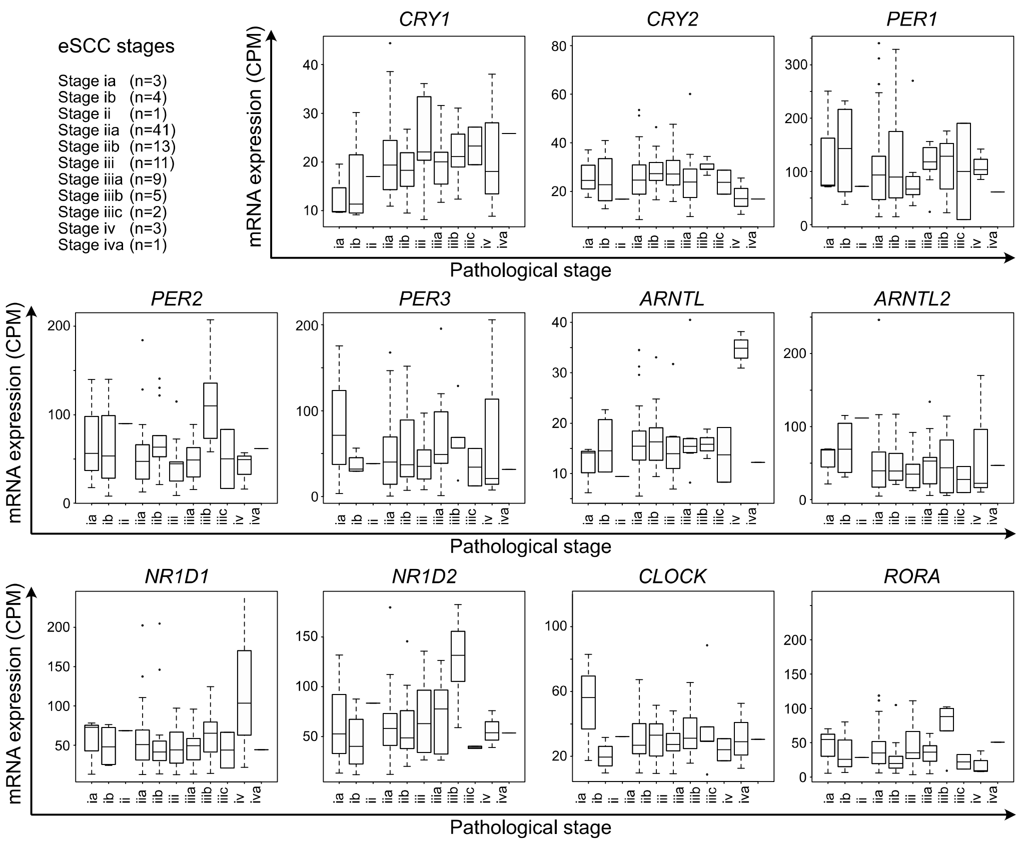

Supplementary figure 1. Expression of clock-related genes in human eSCC samples depending on tumor stages

Expression of *CRY1, CR2, PER1, PER2, PER3, ARNTL, ARNTL2, NR1D1, NR1D2, CLOCK* and *RORA* transcripts measured by RNA sequencing in human eSCC biopsies at different stages of tumor progression. Data are represented as box plots.

Supplementary Figure1

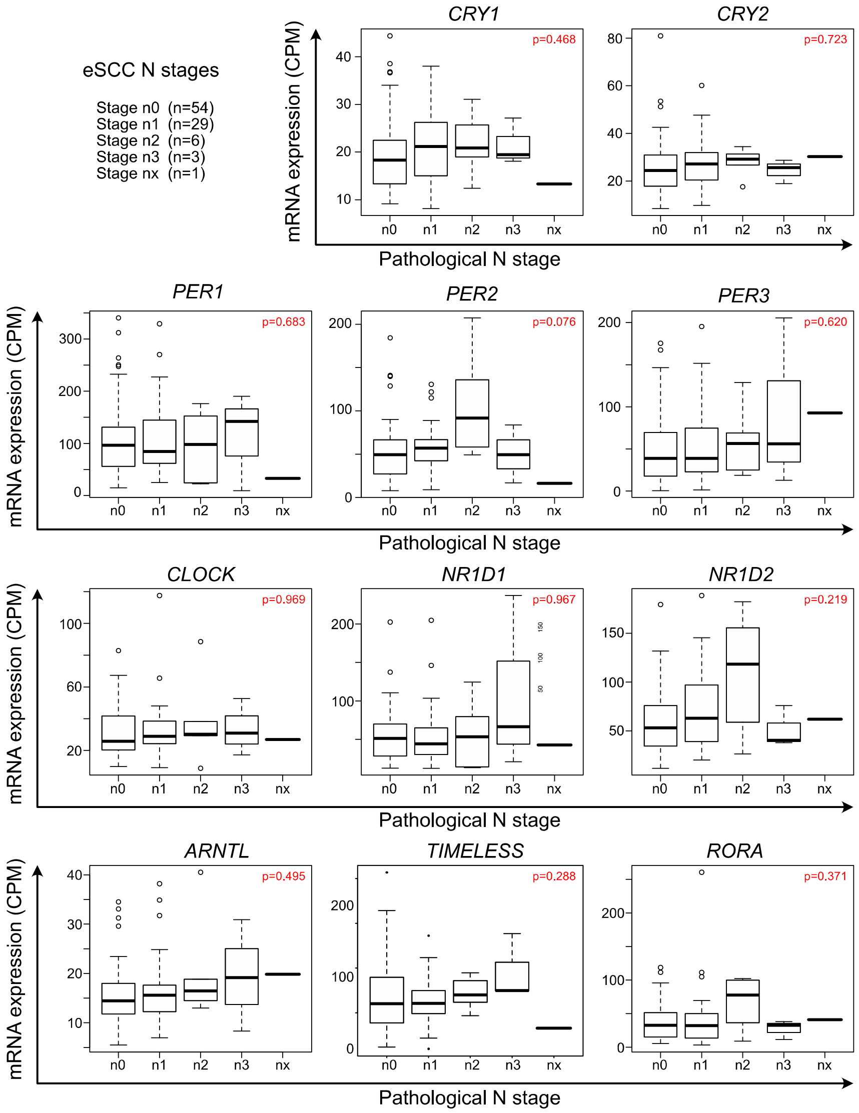

Supplementary figure 2. Expression of clock-related genes in human eSCC samples regarding N tumor pathological stage

Expression of *CRY1, CR2, PER1, PER2, PER3, ARNTL, TIMELESS, NR1D1, NR1D2, CLOCK* and *RORA* transcripts measured by RNA sequencing in human eSCC biopsies at different pathological N stages (lymph node invasion categories). Data are represented as box plots.

Supplementary Figure2

Whole Gel - HSP90

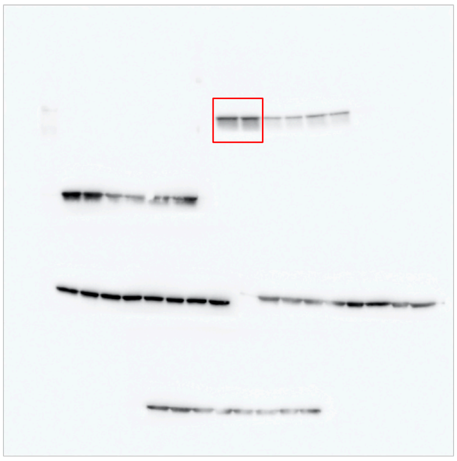

Size marker

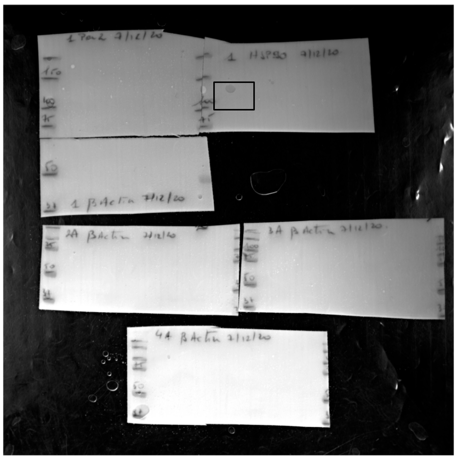

Whole Gel - PER2

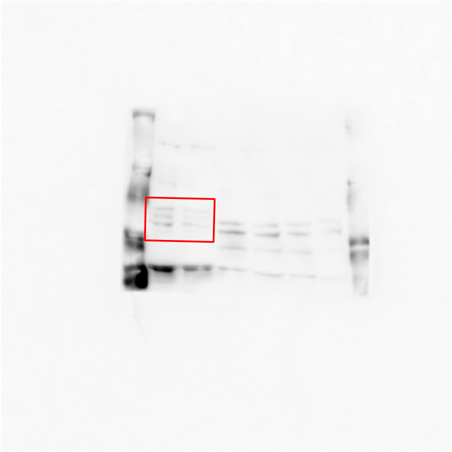

Size marker

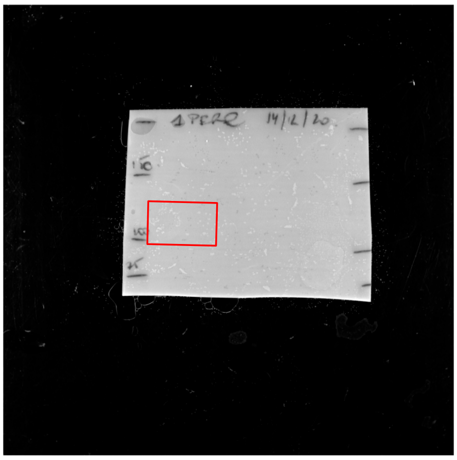

WB	Condition	Image Name	Channel	Name	Signal	Total	Area	Bkgnd.	Type
Per2	24h	20201215_XI	W	00002	154876,969	438078,844	15180	18,6563	Signal
Per2	36h	20201215_XI	W	00001	54874,2813	267394,281	15180	14	Signal
HSP90	24h	20201208_XI	W	00001	127348	144448	2850	6	Signal
HSP90	36h	20201208_XI	W	00003	120844	137944	2850	6	Signal
Normalized I	Condition								
Per/2HSP90	24h	1,21617119							
Per/2HSP90	36h	0,4540919							

Appendix 1

Whole gel - γH2AX

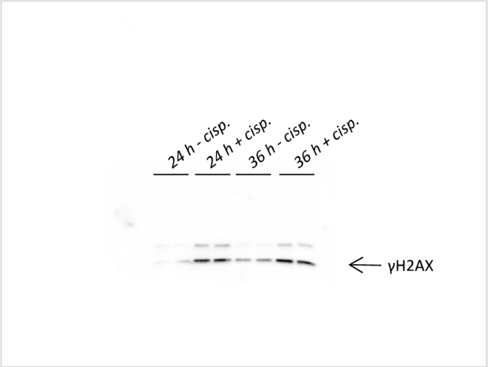

Size marker

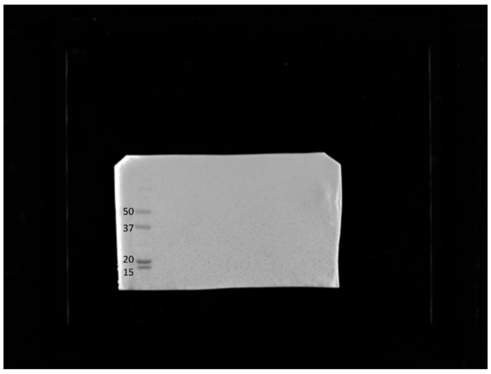

Whole gel - γH2AX + β-actin

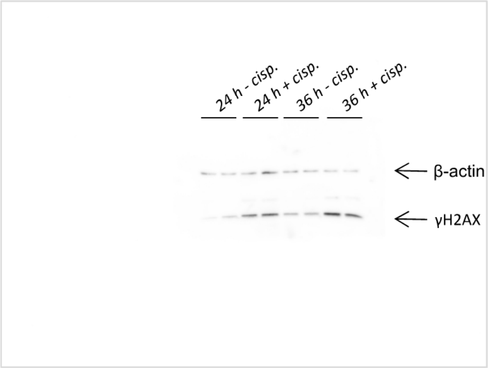

Size marker

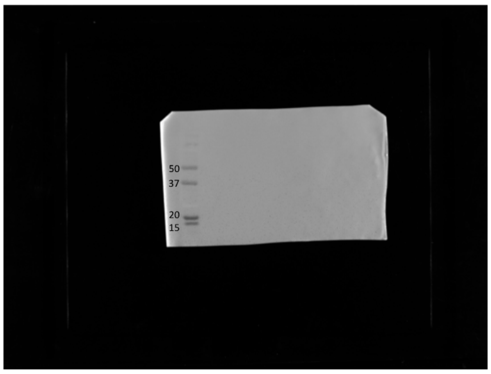

Condition	gH2AX area	b-Actin area	Normalized gH2AX/actin
**24 of sync. Sample 1**	374,213	1817,012	0,206
**24 of sync. Sample 2**	1541,598	1401,477	1,100
**24 of sync. + cisplatin. Sample 1**	4630,74	2344,669	1,975
**24 of sync. + cisplatin. Sample 2**	5767,326	4481,447	1,287
**36 of sync. Sample 1**	2129,719	2236,841	0,952
**36 of sync. Sample 2**	2326,841	1744,305	1,334
**36 of sync. + cisplatin. Sample 1**	8459,64	1322,891	6,395
**36 of sync. + cisplatin. Sample 2**	6219,811	1076,062	5,780
